# Mesoporous Bi_2_S_3_/Bi_2_O_3_ Heterostructure-Based Sensors for Sub-ppm NO_2_ Detection at Room Temperature

**DOI:** 10.3390/s25123612

**Published:** 2025-06-09

**Authors:** Wei Liu, Jiashuo Chen, Ding Gu, Shupeng Sun, Xinlei Li, Xiaogan Li

**Affiliations:** 1 School of Electronics and Information Engineering, Nanjing University of Information Science and Technology, Nanjing 210044, China; liuwei6@nuist.edu.cn (W.L.);; 2 School of Microelectronics and Control Engineering, Changzhou University, Changzhou 213164, China; 3 School of Integrated Circuits, Dalian University of Technology, Dalian 116024, China

**Keywords:** Bi_2_S_3_/Bi_2_O_3_ heterostructures, mesoporous structures, gas sensors, NO_2_ detection, density functional theory

## Abstract

Novel Bi_2_S_3_/Bi_2_O_3_ hybrid materials with unique mesoporous structures were successfully synthesized via a facile in situ elevated-temperature thermal oxidation method using the Bi_2_S_3_ as a precursor in air. The as-prepared Bi_2_S_3_/Bi_2_O_3_ heterostructure-based sensor exhibits an excellent performance for detecting sub-ppm concentrations of NO_2_ at room temperature (RT). In the presence of 8 ppm NO_2_, the sensor registers a response of approximately 7.85, reflecting a 3.5-fold increase compared to the pristine Bi_2_S_3_-based sensor. The response time is 71 s, while the recovery time is 238 s, which are reduced by 32.4% and 24.2%, respectively, compared to the pristine Bi_2_S_3_-based sensor. The Bi_2_S_3_/Bi_2_O_3_ heterostructure-based sensor achieves an impressively low detection limit of 0.1 ppm for NO_2_, and the sensor has been demonstrated to possess superior signal repeatability, gas selectivity, and long-term stability. The optimal preparation conditions of the hybrid materials were explored, and the formation of mesoporous structure was analyzed. The obviously improved gas sensitivity of the Bi_2_S_3_/Bi_2_O_3_ heterostructure-based sensor can be assigned to the combined influence of electronic sensitization and its distinctive morphological structure. The potential gas-sensitive mechanisms were revealed by employing density functional theory (DFT). It was found that the formation of heterostructures could enhance the adsorption energies and increase the amount of electron transfer between NO_2_ molecules and the hybrid materials. Furthermore, the electron redistribution driven by orbital hybridization between O and Bi atoms improves the capacity of NO_2_ molecules to capture additional electrons from the Bi_2_S_3_/Bi_2_O_3_ heterostructures. The content of this work supplies an innovative design strategy for constructing NO_2_ sensor with high performance and low energy consumption at RT.

## 1. Introduction

The threat of air pollution to environmental protection and human health has increased substantially in recent decades, which is caused by the rapid development of industrial automation, leading to the dramatic enhancement in the consumption of traditional energy. As one of the most vital air pollution sources, nitrogen dioxide (NO_2_) is mainly produced by the exhaust emission of fuel vehicles and the combustion of fossil fuels in the industrial scopes [1,2,3]. NO_2_ generates ozone by participating in photochemical reactions, promotes the formation of fine particulate matter (PM2.5), and reacts with water vapor to produce acid rain, which has long-term corrosive effects on vegetation, soil, and water [4,5,6,7]. Moreover, NO_2_ has evident irritation and oxidation. As reported by the World Health Organization, frequent exposure to excessive concentrations of NO_2_ (more than 410 ppb per hour) can induce chronic bronchitis, asthma, pulmonary dysfunction, and even increase the risk of respiratory and cardiovascular diseases [8,9,10]. Therefore, the development of reliable NO_2_ sensors for detecting sub-ppm concentrations of NO_2_ is urgently needed in improving environmental protection and public health.

Elevated operating temperatures are necessary for traditional metal oxide semiconductors (MOSs) due to their insufficient surface activity and sluggish carrier mobility [11]. Long-term exposure to elevated temperatures will accelerate the deterioration of the performance of MOSs-based sensors and decrease the reliability and service lifetimes of the sensors. Furthermore, the supplementary energies will increase the complexity and energy consumption of the sensing system, which inhibits the practical application in special occasions such as flammable and explosive [12,13]. Compared to the MOSs, two-dimensional (2D) materials have garnered extensive attention from researchers and have gradually emerged as the research hotspot within the scope of gas sensors owing to their large specific surface area, adjustable band structures, high carrier mobility, and rich active sites [14,15,16,17]. Meanwhile, quasi-2D sulfides, including TiS_3_, SnS_2_, and WS_2_, have also been proven to possess excellent gas detection capabilities due to their layered structure and chemical properties similar to those of 2D materials [18,19,20]. As a typical 2D material, Bi_2_S_3_ has been widely investigated for gas detection because of its unique physicochemical, optical, and electrical properties [21,22,23,24,25]. For instance, Harke et al. proved that the Bi_2_S_3_ nanostructured film-based sensor fabricated by the solvothermal process exhibited outstanding selectivity toward 100 ppm NO_2_ in the interfering gases of NH_3_, CO_2_, C_2_H_5_OH, and H_2_S [21]. Russ et al. revealed that the synthesized Bi_2_S_3_ nanowire-based sensor showed reliable long-term stability to 3 ppm NO_2_ within 42 days, and the average relative error of the sensor was less than 10.5% [22]. Yang et al. demonstrated that the Bi_2_S_3_-based sensor with a nanorod structure has superior sensitivity for detecting 1 ppm NO_2_ at RT, making it a potential material for developing high-performance gas sensors [23]. Nonetheless, the inherent limitations of Bi_2_S_3_, involving the inadequate sensitivity and the poor signal repeatability, restrict its application in detecting sub-ppm concentrations of NO_2_ at RT.

Constructing hybrid materials is regarded as a productive strategy to address the limitations of single-component sensitive materials, which has been thoroughly explored to enhance the gas-sensitive characteristics of the sensors. The formed heterostructures could improve the transmission and accumulation of carriers, promote the adsorption and desorption of gas molecules, and increase the interaction efficiency between gas molecules and hybrid materials due to the electron sensitization caused by the built-in electric field [26,27]. Thus, compared to the single-component sensitive materials such as pristine Bi_2_S_3_, the hybrid materials could exhibit excellent sensitivity and signal repeatability [28,29,30]. Moreover, the accurate regulation of the morphological structure of hybrid materials could further increase the sensitivity of the sensor due to the enlargement of specific surface area, which is conducive to the adsorption of abundant gas molecules [31,32]. However, the improvement of gas-sensitive performance of the hybrid Bi_2_S_3_-based sensor with controllable morphological structure has rarely been reported, and the sensitization mechanisms still need further exploration.

In this work, we report a novel chemiresistive-type sensor capable of detecting sub-ppm levels of NO_2_ at RT, which employed the Bi_2_S_3_/Bi_2_O_3_ heterostructures with mesoporous as the sensitive materials. Due to the influence of electron sensitization, the transfer of carriers on the surface of Bi_2_S_3_/Bi_2_O_3_ hybrid materials and the adsorption/desorption process of NO_2_ molecules are substantially improved. Moreover, the unique mesoporous structure offers massive active sites and facilitates the deep diffusion behavior of NO_2_ molecules; thus, the gas-sensitive performance of the prepared sensor could be substantially enhanced.

## 2. Experimental Section

### 2.1. Fabrication of Mesoporous Bi_2_S_3_/Bi_2_O_3_ Hybrid Materials

**Figure 1:**
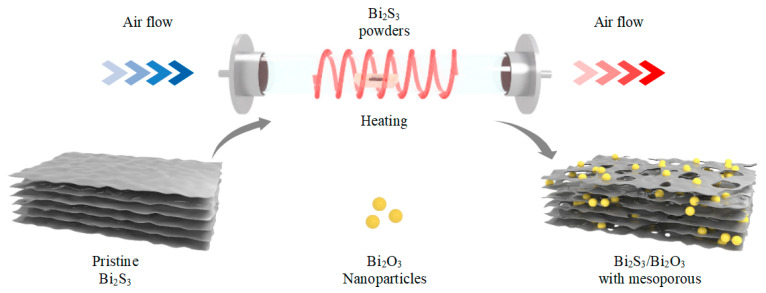
Schematic diagram of the fabrication process of Bi_2_S_3_/Bi_2_O_3_ hybrid materials with mesoporous structure.

The Bi_2_S_3_/Bi_2_O_3_ hybrid materials with mesopores were fabricated by one-step in situ elevated temperature thermal oxidation in air, as illustrated in Figure 1. Firstly, 200 mg Bi_2_S_3_ powders (Chengdu Alfa Metal Materials Co., Ltd., Chengdu, China, 99.999%) were evenly dispersed in an alumina ceramic boat inside a tube furnace (OTF-1200X-S, Hefei Kejing of MTI Corp, Hefei, China). Subsequently, the synthetic air (200 sccm/min) was introduced into the tube furnace, and the thermal oxidation was performed at temperatures ranging from 300~500 °C. The powders were heated to the desired temperature at the rate of 3 °C/min, and the temperature was maintained for 0~2 h, respectively. After the oxidation procedure, the powders were naturally cooled down to RT. Finally, the Bi_2_S_3_/Bi_2_O_3_ hybrid materials prepared at different temperatures and oxidation times were obtained.

### 2.2. Material Characterizations

The thermostability of the Bi_2_S_3_ powders was characterized using a thermogravimetric analyzer (TGA, STA200, Hitachi Ltd., Tokyo, Japan) at a thermal ramp rate of 5 °C/min in air. The crystallographic information of the synthesized materials was identified utilizing X-ray diffraction (XRD, XRD-6000, PANalytical B.V., Almelo, Netherlands) with Cu Kα radiation. The morphological structures of the hybrid materials were recorded by scanning electron microscopy (SEM, Regulus8100, Hitachi Ltd., Tokyo, Japan) and transmission electron microscopy (TEM, Talos F200X G2, FEI Company, Hillsboro, OR, USA). The elemental states were investigated by X-ray photoelectron spectroscopy (XPS, Escalab250Xi, Thermo Fisher Scientific, Waltham, MA, USA). The pore diameter distributions and specific surface area of the hybrid materials were verified using Brunauer–Emmett–Teller (BET, NOVA2000e, Quantachrome Instruments, Boynton Beach, FL, USA). The elemental defects in the hybrid materials were measured through an electron paramagnetic resonance spectrometer (EPR, JES-FA200, JEOL Ltd., Tokyo, Japan). The work functions of the hybrid materials were confirmed by Kelvin probe force microscopy (KPFM, SKP5050, KP Technology, Wick, UK).

### 2.3. Sensor Preparation and Measurements

In a typical synthesis procedure, 5 mg of the as-prepared Bi_2_S_3_/Bi_2_O_3_ hybrid materials were placed into an agate mortar. Subsequently, a small amount of deionized water (~0.1 mL) was added, and the mixture was thoroughly ground for 10 min to form a printable paste. The paste was then uniformly dispensed onto the alumina substrate precoated with Au interdigitated electrodes (Changchun Beirun Electronics Technology Co., Ltd., Changchun, China, 10 × 5 × 0.635 mm, 8 pairs of electrodes, finger spacing of 200 μm, finger width of 200 μm) using a pipette gun (DLAB Scientific Co., Ltd., Beijing, China), achieving a thickness of approximately 3 μm.

The Bi_2_S_3_/Bi_2_O_3_ heterostructure-based sensors were developed by transferring the interdigital electrodes into a vacuum oven (DZF-6020, Hefei Kejing of MTI Corp, Hefei, China) and drying them at 60 °C for 12 h.

The measurement platform consisted of a digital signal detection system (Agilent 34410A, Santa Clara, CA, USA) and a dynamic gas allocation system (Beijing Sevenstar Huachuang Meter Co., Ltd., Beijing, China). The digital signal detection system used the Agilent 34410A electrometer to register the resistance of the sensors. The dynamic gas allocation system employed the Mass Flow Controllers (MFCs) to dilute the purchased 10 ppm NO_2_ to different concentrations by accurately controlling the flow rate of synthetic air. The humidity in the reaction chamber was regulated by humidifying synthetic air with different saturated salt solutions. The sensor response (S) was determined by the ratio of the saturated resistance in the measured gases ($R_{gas}$) to that in the background gas ($R_{air}$, synthetic air: O_2_/N_2_ = 1/8), as expressed by the following formulas:

(1) $$S=R_{gas}/R_{air}\;(\mathrm{for}\;\mathrm{oxidizing}\;\mathrm{gases})$$

(2) $$S=R_{air}/R_{gas}\;(\mathrm{for}\;\mathrm{reducing}\;\mathrm{gases})$$

The response/recovery time was calculated by measuring when the sensor resistance reached/recovered 90% of the saturated value.

### 2.4. DFT Computation

Theoretical computations were conducted using the Vienna Ab-initio Simulation Package (VASP.5.4.4) to analyze the adsorption energy, electron transfer amount, and DOS for NO_2_ molecules interacting with Bi_2_S_3_/Bi_2_O_3_ hybrid materials. The Perdew-Burke-Ernzerhof (PBE) of generalized gradient approximation (GGA) was employed for the exchange-correlation pseudopotential [33,34,35]. The influence of van der Waals interactions was corrected employing the DFT-D3 method [36,37]. The Brillouin zones were sampled using 2 × 2 × 1 and 9 × 9 × 1 k-points for structure optimization and DOS calculation, respectively. A force convergence criterion of 0.05 eV/Å and a plane-wave cutoff energy of 450 eV were utilized. The energy convergence standard of structural self-consistency was set to 1 × 10^−5^ eV/atom. Additionally, a 15 Å vacuum layer was included to prevent interactions between adjacent units. The adsorption energy ($E_{ads}$) was described as given below.

(3) $$E_{ads}=E_{sub+gas}-E_{sub}-E_{gas}$$

where $E_{sub+gas}$, $E_{sub}$, and $E_{gas}$ represent the total energy of the gas adsorption configuration, the energy of the Bi_2_S_3_/Bi_2_O_3_ heterostructure, and the energy of the NO_2_ molecule, respectively. The negative value of $E_{ads}$ indicates that the interaction is exothermic, and the greater the |$E_{ads}$|, the stronger the stability of NO_2_ adsorption.

## 3. Results and Discussion

### 3.1. Microstructure of Bi_2_S_3_/Bi_2_O_3_ Hybrid Materials

**Figure 2:**
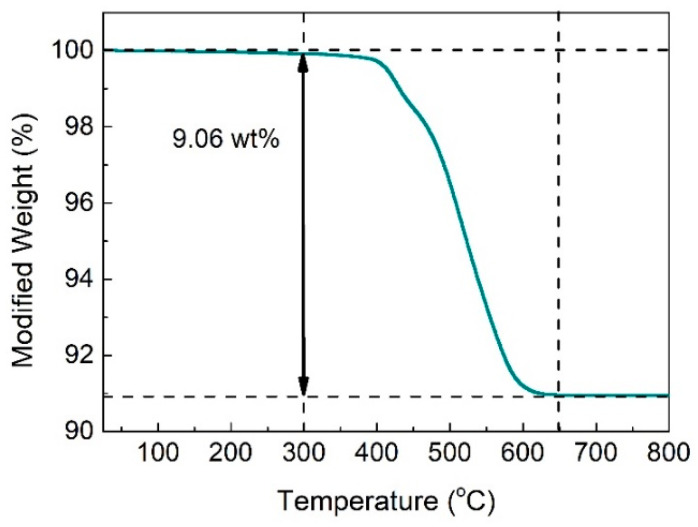
TGA curve of the pristine Bi_2_S_3_ powders from 25 °C to 800 °C in air.

The thermal stability of the pristine Bi_2_S_3_ was investigated by TGA from 25 °C to 800 °C with a ramping rate of 10 °C/min in air. As shown in Figure 2, the pristine Bi_2_S_3_ was relatively stable below 300 °C with only about 0.07% weight loss, which could be assigned to the evaporation of pre-adsorbed water molecules. In the temperature range of 300 °C to 650 °C, the weight of Bi_2_S_3_ obviously decreased by approximately 9.06% (theoretical weight loss was calculated to be 9.37%), indicating that the redox reaction occurred between Bi_2_S_3_ and O_2_. The weight loss is caused by the direct volatilization of an abundance of SO_2_ produced during the following reaction:

(4) $$2{Bi}_{2}S_{3}+9O_{2}=2{Bi}_{2}O_{3}+6{SO}_{2}$$

There was no obvious weight loss between 650 °C and 800 °C, illustrating that the Bi_2_S_3_ had been completely oxidized to Bi_2_O_3_ in this temperature range. Therefore, according to the analysis results of the thermal stability of pristine Bi_2_S_3_, the Bi_2_S_3_/Bi_2_O_3_ hybrid materials were fabricated utilizing 300 °C, 350 °C, 400 °C, 450 °C, and 500 °C as oxidation temperatures and controlling the oxidation times at 0 h, 0.5 h, 1 h, and 2 h, respectively.

**Figure 3:**
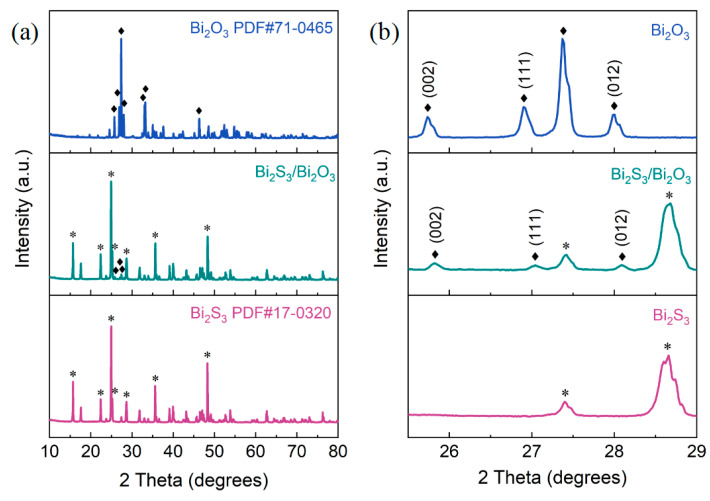
(**a**) XRD patterns of the pristine Bi_2_S_3_, Bi_2_S_3_/Bi_2_O_3_ hybrid materials, and Bi_2_O_3_. (**b**) An enlarged view of the selected region of XRD.

The crystal structure and crystal-phase information of the pristine Bi_2_S_3_, Bi_2_S_3_/Bi_2_O_3_ hybrid materials, and Bi_2_O_3_ were characterized by XRD. The Bi_2_S_3_/Bi_2_O_3_ hybrid materials were prepared at 400 °C for 1 h, and the Bi_2_O_3_ was obtained at 700 °C for 1 h under the same fabrication method. In Figure 3a, the diffraction peaks located at 15.74°, 24.84°, 25.34°, 35.71°, and 48.36° are indexed to the (020), (130), (310), (240), and (060) lattice planes of Bi_2_S_3_ (JCPDS No. 17-0320), respectively, illustrating that the pristine Bi_2_S_3_ possesses an orthogonal phase [38,39]. Similarly, the diffraction peaks at 25.71°, 26.97°, 27.36°, 32.93°, and 33.31° are assigned to the (002), (111), (120), (121), and (200) crystal planes of Bi_2_O_3_ (JCPDS No. 71-0465) with a monoclinic structure [40,41]. As shown in Figure 3b, the three weak peaks of Bi_2_S_3_/Bi_2_O_3_ at 2θ of 25.84°, 27.05°, and 28.09°, which could be attributed to the (002), (111), and (012) planes of Bi_2_O_3_, demonstrate that the Bi_2_O_3_ nanoparticles were formed during the elevated temperature thermal oxidation process. It should be noted that, compared to the Bi_2_O_3_, the positions of the three diffraction peaks are slightly shifted by approximately 0.1° towards the large-angle direction, implying that the introduction of the O element causes the lattice distortion, and the lattice spacing of Bi_2_S_3_ becomes smaller [42,43].

**Figure 4:**
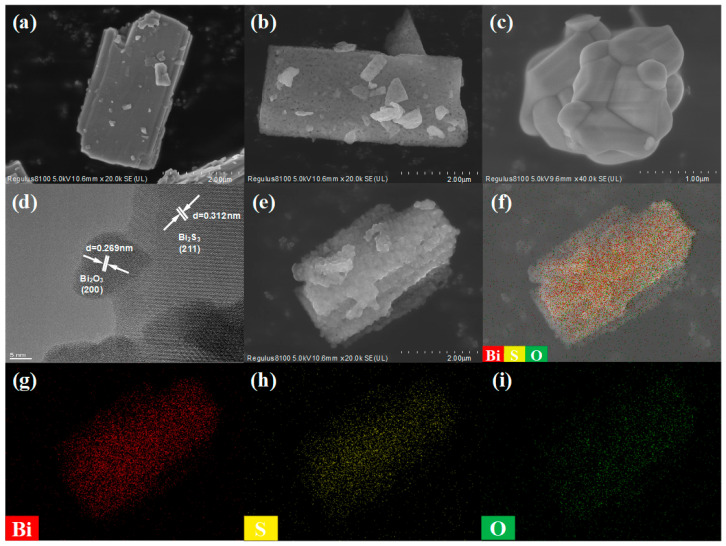
(**a**–**c**) SEM images of the pristine Bi_2_S_3_, Bi_2_S_3_/Bi_2_O_3_ hybrid materials, and Bi_2_O_3_; (**d**) HRTEM image of Bi_2_S_3_/Bi_2_O_3_; and (**e**–**i**) elemental mapping of Bi_2_S_3_/Bi_2_O_3_.

The morphological evolution procedure of the as-synthesized materials prepared at various temperatures was investigated by SEM and TEM, as described in Figure 4. In Figure 4a, the surface of the pristine Bi_2_S_3_ microflake without elevated-temperature thermal oxidation treatment was smooth, showing the typical layered structure characteristic of 2D materials, and the length was in the range of 4~6 μm. As the temperature increased, the surface of the materials became rough gradually. Notably, compared to the pristine Bi_2_S_3_, the surface of Bi_2_S_3_/Bi_2_O_3_ hybrid materials prepared at 400 °C for 1 h exhibits abundant mesoporous structures, as shown in Figure 4b. This structural feature is beneficial to enhance the specific surface area of the hybrid materials and promote the deep diffusion of gas molecules, which is required by chemiresistive-type semiconductor gas sensors. Figure 4c displays the Bi_2_O_3_ obtained at 700 °C for 1 h. It could be observed that the previously formed mesoporous structures had disappeared, and the surface of Bi_2_O_3_ had changed to be smoother and denser. This could be due to the decrease in surface energy with the progress of redox reaction [44]. In accordance with the principle of surface energy minimization, the redistribution of surface atoms reduces the number of surface defects and dangling bonds with higher energy, thus altering the morphology of Bi_2_O_3_. Figure 4d illustrates the high-resolution TEM image of the Bi_2_S_3_/Bi_2_O_3_ hybrid materials fabricated at 400 °C for 1 h. It can be seen that a Bi_2_O_3_ nanoparticle with a diameter of about 10 nm was formed at the edge of the Bi_2_S_3_ microflake. The 0.312 nm lattice spacing corresponds to the (211) crystalline plane of Bi_2_S_3_, while the 0.269 nm lattice spacing is consistent with the (200) crystalline plane of Bi_2_O_3_, implying the development of a Bi_2_S_3_/Bi_2_O_3_ heterostructure. The elemental mapping images of the Bi_2_S_3_/Bi_2_O_3_ hybrid materials are presented in Figure 4e–i. The elements of Bi (red), S (yellow), and O (green) are homogeneously and continuously distributed, demonstrating that the Bi_2_O_3_ nanoparticles are uniformly spread on the surface of Bi_2_S_3_ microflakes without agglomeration. This makes the active sites completely exposed, which improves the sensitivity and response speed of the Bi_2_S_3_/Bi_2_O_3_ heterostructure-based sensors.

**Figure 5:**
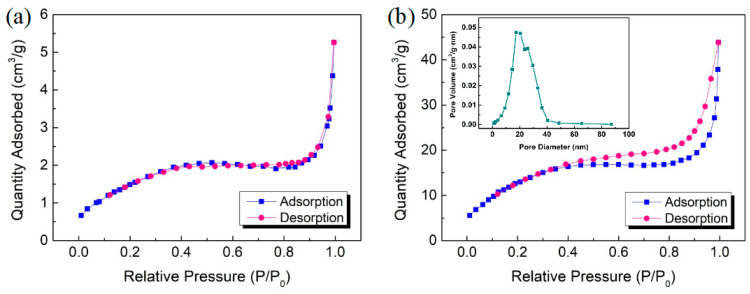
N_2_ adsorption/desorption isotherms and pore size distribution of (**a**) pristine Bi_2_S_3_ and (**b**) Bi_2_S_3_/Bi_2_O_3_ hybrid materials.

In Figure 5, the N_2_ adsorption/desorption isotherms and pore size distribution were characterized to determine the specific surface area and porosity of the hybrid materials synthesized with the introduction of Bi_2_O_3_ nanoparticles. The curves of the pristine Bi_2_S_3_ and the Bi_2_S_3_/Bi_2_O_3_ hybrid materials prepared at 400 °C for 1 h both exhibit the typical type-IV isotherms. In Figure 5a, in the range of relative pressure ($P/P_{0}$) from 0.0 to 1.0, no hysteresis loop could be observed in the pristine Bi_2_S_3_. In contrast, a hysteresis loop appeared obviously within the range of 0.4~1.0 ($P/P_{0}$) for the hybrid materials, which could be assigned to the unique mesoporous structure on the surface of Bi_2_S_3_/Bi_2_O_3_, as illustrated in Figure 5b. The pore size distribution curve of Bi_2_S_3_/Bi_2_O_3_ demonstrates that the average pore size is about 17.4 nm. Furthermore, the specific surface areas of pristine Bi_2_S_3_ and the Bi_2_S_3_/Bi_2_O_3_ hybrid materials were recorded to be 3.31 m^2^g^−1^ and 26.92 m^2^g^−1^, respectively. It could be found that the mesoporous structure was formed on the surface of hybrid materials through in situ elevated temperature thermal oxidation, and the specific surface area was significantly improved by approximately 8.1-fold. This desired structure supplies additional adsorption sites, which enhances the ability to attract gas molecules.

**Figure 6:**
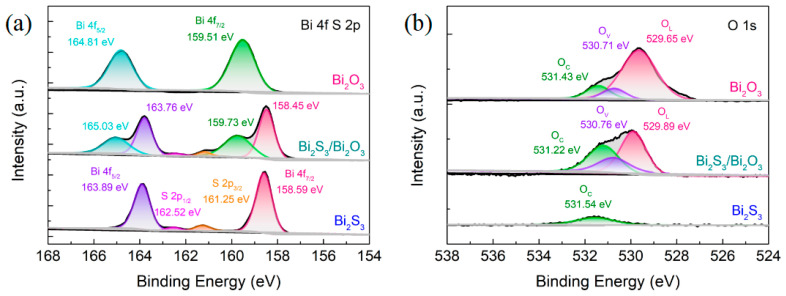
XPS spectra of the pristine Bi_2_S_3_, Bi_2_S_3_/Bi_2_O_3_ hybrid materials, and Bi_2_O_3_ in the (**a**) Bi 4f and S 2p regions and (**b**) O 1s region.

The chemical states of surface elements of pristine Bi_2_S_3_, Bi_2_S_3_/Bi_2_O_3_ hybrid materials, and Bi_2_O_3_ were identified by XPS measurement. The survey spectrums (see Figure S1) demonstrate that Bi, S, and O elements coexist in the Bi_2_S_3_/Bi_2_O_3_ hybrid materials prepared at 400 °C for 1 h, consistent with the elemental mapping results. After elevated-temperature thermal oxidation at 700 °C for 1 h, no S 2s elemental peak could be detected in Bi_2_O_3_, illustrating that the pristine Bi_2_S_3_ had been completely oxidized to Bi_2_O_3_. Figure 6a describes the XPS spectra of Bi 4f and S 2p in the synthesized materials. In the pristine Bi_2_S_3_, the elemental peaks at 158.59 eV and 163.89 eV correspond to Bi 4f_7/2_ and Bi 4f_5/2_, respectively [28]. Similarly, in Bi_2_O_3_, these peaks are found at 159.51 eV and 164.81 eV [45]. It reveals that the valence of bismuth atoms is Bi^3+^ in both pristine Bi_2_S_3_ and Bi_2_O_3_. In Bi_2_S_3_/Bi_2_O_3_ hybrid materials, the elemental peaks of Bi 4f are split into two groups, and the peaks centered at 158.45 eV and 163.76 eV correspond to Bi-S bonds, while those at 159.73 eV and 165.03 eV are considered to be Bi-O bonds [46]. It could be observed that, compared to the pristine Bi_2_S_3_ and Bi_2_O_3_, the peaks of Bi-S bonds shifted upward by about 0.14 eV toward the lower binding energy, while the peaks of Bi-O bonds shifted downward by approximately 0.22 eV in the opposite direction. This could be assigned to the transfer and accumulation of carriers at the heterostructure interfaces due to the built-in electric field [47,48]. Moreover, the elemental peaks of S 2p occurred at 161.25 eV and 162.52 eV and are assigned to the S 2p_3/2_ and S 2p_1/2_, implying that the valence of sulfur atoms is S^2−^ in both pristine Bi_2_S_3_ and Bi_2_S_3_/Bi_2_O_3_ hybrid materials [49].

Figure 6b shows the O 1s spectrums of the as-synthesized materials. The elemental peak at 531.54 eV in the pristine Bi_2_S_3_ corresponds to the chemisorbed oxygen ions (O_C_), and the species should be $O_{2}^{-}$ at RT [50,51]. The O 1s spectrum in the Bi_2_S_3_/Bi_2_O_3_ hybrid materials displays three peaks at 531.22 eV, 530.76 eV, and 529.89 eV, which could be assigned to the O_C_, oxygen vacancies (O_V_), and lattice oxygen ions (O_L_) [52,53]. The binding energies of O_C_, O_V_, and O_L_ in Bi_2_O_3_ shift to 531.43 eV, 530.71 eV, and 529.65 eV, respectively, as the oxidation temperature rises [54,55]. By calculating the integral area of each peak in the O 1s spectrum, the relative concentrations of O_C_, O_V_, and O_L_ in the prepared materials can be determined and summarized (see Table S1). It could be found that compared to the pristine Bi_2_S_3_, the content of O_C_ in Bi_2_S_3_/Bi_2_O_3_ hybrid materials is significantly enhanced by approximately 3.05-fold. However, the improvement of the content of O_C_ in Bi_2_O_3_ is only 1.13-fold. This could be due to the formation of heterostructures promoting the adsorption of oxygen molecules on the surface of hybrid materials. In addition, the proportion of O_V_ in Bi_2_S_3_/Bi_2_O_3_ and Bi_2_O_3_ was estimated to be about 25.27% and 11.29%, respectively. These oxygen vacancies act as capture centers of O_2_ molecules, providing additional active sites for the gas-sensitive process.

**Figure 7:**
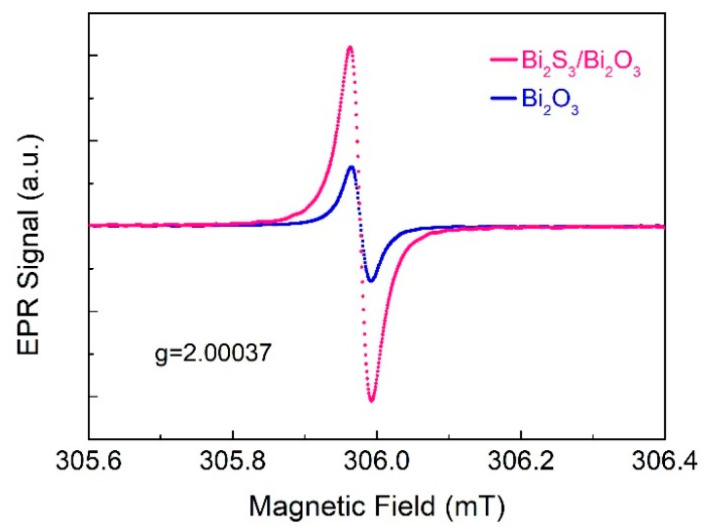
EPR spectra of the Bi_2_S_3_/Bi_2_O_3_ hybrid materials and Bi_2_O_3_.

EPR was employed to investigate the oxygen vacancies in the Bi_2_S_3_/Bi_2_O_3_ hybrid materials prepared at 400 °C for 1 h and in the Bi_2_O_3_ obtained at 700 °C for 1 h. In Figure 7, the microwave frequency was set to 9.45 GHz, and the EPR signal at g = 2.00037 confirmed oxygen vacancies in both Bi_2_S_3_/Bi_2_O_3_ and Bi_2_O_3_ [56]. Compared to the Bi_2_O_3_, the EPS signal intensity of Bi_2_S_3_/Bi_2_O_3_ is higher, demonstrating that the unpaired electrons in the hybrid materials present more pronounced interaction with the external magnetic field. It reveals that the oxygen vacancy content in Bi_2_S_3_/Bi_2_O_3_ hybrid materials exceeds that in Bi_2_O_3_, consistent with XPS analysis.

### 3.2. Gas-Sensitive Properties

**Figure 8:**
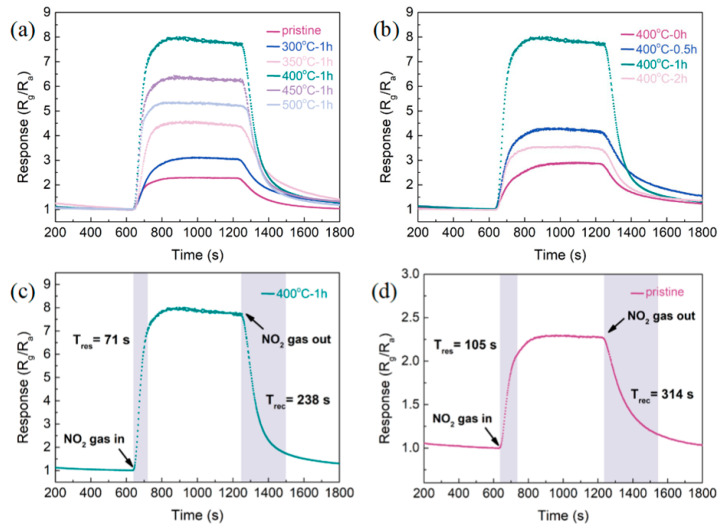
Response curves of the Bi_2_S_3_-based sensor and Bi_2_S_3_/Bi_2_O_3_ heterostructure-based sensors to 8 ppm NO_2_ under various (**a**) oxidation temperatures and (**b**) oxidation times, and the response/recovery time of (**c**) the Bi_2_S_3_/Bi_2_O_3_ heterostructure-based sensor and (**d**) the Bi_2_S_3_-based sensor.

To determine the gas-sensitive performance of the sensors under various preparation conditions, the response curves of the pristine Bi_2_S_3_-based sensor and the Bi_2_S_3_/Bi_2_O_3_ heterostructure-based sensors fabricated at different thermal oxidation temperatures (from 300 °C to 500 °C) and oxidation times (from 0 h to 2 h) to 8 ppm NO_2_ are shown in Figure 8a,b. In Figure 8a (with oxidation time fixed at 1 h), the response curves of all synthesized sensors exhibited an upward trend when NO_2_ was introduced into the reaction chamber, and the curves showed the opposite behavior after NO_2_ was removed. Since NO_2_ is a typical oxidizing gas species, combined with Formula (1), it could be found that the prepared Bi_2_S_3_/Bi_2_O_3_ hybrid materials take electrons as the majority carriers, indicating the characteristics of N-type semiconductors. Furthermore, with the oxidation temperature increased from 300 °C to 350 °C, the responses rose from 3.08 to 4.52, which illustrated that the sensitivity of the sensors to NO_2_ had been enhanced gradually. When the oxidation temperature reached 400 °C, the Bi_2_S_3_/Bi_2_O_3_ heterostructure-based sensor exhibited a response of approximately 7.85 to 8 ppm NO_2_, achieving the maximum response value. This response surpassed that of the pristine Bi_2_S_3_-based sensor by 3.5-fold. However, the sensing characteristics of the sensors started to deteriorate as the oxidation temperature rose from 450 °C to 500 °C, with the responses decreasing to 6.29 and 5.31, respectively. The decline of gas-sensitive performance could be related to the reduction in heterostructure content. Thus, the optimal oxidation temperature was determined to be 400 °C. Notably, the resistance of the Bi_2_O_3_-based sensor prepared at 700 °C exceeded the measurement range of the digital multimeter at RT (~1200 MΩ), rendering it impossible to obtain its response curve to NO_2_. The I-V curves (see Figure S2) of the pristine Bi_2_S_3_, the Bi_2_S_3_/Bi_2_O_3_ heterostructure, and the Bi_2_O_3_-based sensors present a linear relationship (Ohmic contact) at RT, indicating adequate contact between the sensitive materials and the interdigital electrodes.

Figure 8b depicts the impact of various oxidation times on the characteristics of the prepared sensors at a constant oxidation temperature of 400 °C. It could be observed that the response of Bi_2_S_3_/Bi_2_O_3_ heterostructure-based sensors increased initially and then decreased as the oxidation time rose from 0 h to 2 h. The optimal oxidation time is distinctly identified as 1 h. In Figure 8c,d, the response and recovery times of the Bi_2_S_3_/Bi_2_O_3_ heterostructure-based sensor and the pristine Bi_2_S_3_-based sensor have been calculated. In comparison to the pristine Bi_2_S_3_-based sensor, the Bi_2_S_3_/Bi_2_O_3_ heterostructure-based sensor produced at 400 °C for 1 h exhibited response and recovery times of 71 s and 238 s, which were reduced by approximately 32.4% and 24.2%, respectively. Obviously, the construction of the heterostructure could significantly enhance the sensitivity and the response/recovery speed of the pristine Bi_2_S_3_-based sensor.

**Figure 9:**
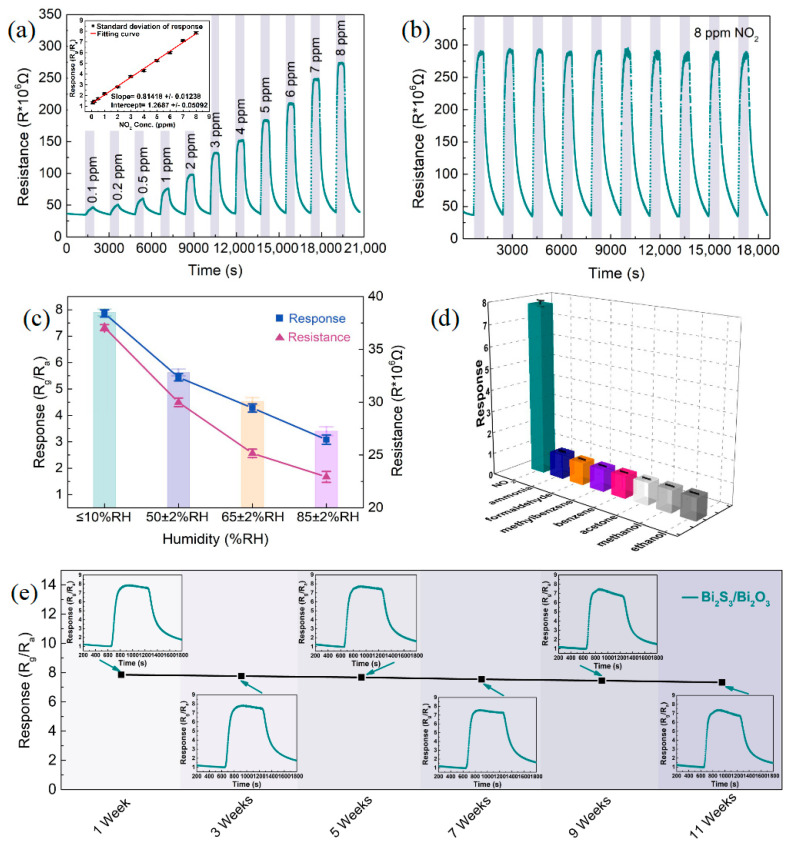
(**a**) Resistance curve of the Bi_2_S_3_/Bi_2_O_3_ heterostructure-based sensor to NO_2_ concentrations from 0.1 ppm to 8 ppm (inset describes the corresponding response fitting curve), (**b**) signal repeatability, (**c**) response and resistance of the sensor under various humidity conditions, (**d**) selectivity, and (**e**) long-term stability to 8 ppm NO_2_.

Figure 9a displays the resistance curve of the Bi_2_S_3_/Bi_2_O_3_ heterostructure-based sensor obtained utilizing the optimal oxidation temperature (400 °C) and oxidation time (1 h) for detecting NO_2_ at concentrations ranging from 0.1 ppm to 8 ppm. As the concentration of NO_2_ increased continuously, the resistance variation scope of the sensor expanded gradually. The initial resistance was approximately 36 MΩ, which could be recovered completely after the termination of NO_2_; no drift of the baseline resistance was observed. The response fitting curve in the illustration (inset) reveals that the response of the Bi_2_S_3_/Bi_2_O_3_ heterostructure-based sensor had a linear correlation with the change of NO_2_ concentration, and the sensitivity of the sensor was calculated to be 0.81418 ± 0.01238 ppm^−1^. Figure 9b shows the signal repeatability verification of the Bi_2_S_3_/Bi_2_O_3_ heterostructure-based sensor. The results of 10 cyclic measurements of 8 ppm NO_2_ confirmed that the response deviation of the synthesized sensor was less than 3.4%, and it exhibited excellent signal repeatability. As we know, environmental humidity has an outstanding influence on the performance of chemiresistive-type gas sensors. Thus, the sensing properties of the Bi_2_S_3_/Bi_2_O_3_ heterostructure-based sensor were examined at varying humidities, as depicted in Figure 9c. The response and resistance of the Bi_2_S_3_/Bi_2_O_3_ heterostructure-based sensor decreased gradually as the ambient humidity rose (corresponding curves see Figures S3 and S4). At 85% ± 2% RH, the response of the sensor was only 39.14% (~3.08) of that in the low-humidity environment (≤10% ± 2% RH). This could be assigned to water molecules in high-humidity environments occupying the active sites on the surface of Bi_2_S_3_/Bi_2_O_3_ hybrid materials, which weakens the contact and reaction opportunities between gas molecules and hybrid materials, resulting in the deterioration of the surface activity of the sensor. Meanwhile, the reduction in chemisorbed oxygen ions decreases the surface potential of Bi_2_S_3_/Bi_2_O_3_ hybrid materials. This increase in conductivity covered the resistance change caused by NO_2_ molecules, thus decreasing the response of the sensor.

Figure 9d describes the response of the Bi_2_S_3_/Bi_2_O_3_ heterostructure-based sensor to 8 ppm NO_2_ and 100 ppm concentrations of various interfering gases, including ammonia, formaldehyde, methylbenzene, benzene, acetone, methanol, and ethanol. Clearly, compared to the interfering gas species, the as-prepared sensor exhibited a significantly stronger interaction with low concentrations of NO_2_, proving its superior gas selectivity. The long-term stability of the Bi_2_S_3_/Bi_2_O_3_ heterostructure-based sensor was evaluated as described in Figure 9e. During the entire verification process, the sensor was assessed every two weeks, and it was stored in the natural environment for the rest of the time. The slight decrease in sensor response in the initial stage could be related to the aging of Bi_2_S_3_/Bi_2_O_3_ hybrid materials. This procedure could partially passivate the dangling bonds on the surface of the hybrid materials, thus reducing the number of defect states. In spite of this, after 11 weeks of measurement, the response deviation of the sensor to 8 ppm NO_2_ remained less than 6.6%, and the responses tended to stabilize gradually, demonstrating its outstanding long-term stability.

The endurance of the sensor is a vital criterion for assessing its stable operation in complex environments and plays a crucial role in ensuring the accuracy and reliability of gas detection. According to the above experimental results, compared to its anti-humidity interference ability, the Bi_2_S_3_/Bi_2_O_3_ heterostructure-based sensor fabricated in this paper exhibits remarkable signal repeatability, gas selectivity, and long-term stability. This makes it comply with the practical application requirements in various harsh scenarios under moderate and low-humidity conditions. Thus, the developed Bi_2_S_3_/Bi_2_O_3_ hybrid materials are expected to be a high-performance candidate for the detection of trace NO_2_.

The gas-sensitive characteristics of the Bi_2_S_3_/Bi_2_O_3_ heterostructure-based sensor developed in this work for detecting NO_2_ at RT were compared to those of Bi_2_S_3_-based sensors reported in the literature, as summarized in Table 1. It can be concluded that the specific surface area of the synthesized Bi_2_S_3_/Bi_2_O_3_ hybrid materials was increased by constructing the heterostructure and combining it with the unique mesoporous structure. This provided more adsorption sites for NO_2_ molecules, significantly improving the detection performance of Bi_2_S_3_-based sensors. Therefore, the as-prepared sensor possesses superior sensitivity, rapid response and recovery speed, and a lower detection limit for sensing low-concentration NO_2_.

### 3.3. Sensitization Mechanisms

#### 3.3.1. Roles of Bi_2_S_3_/Bi_2_O_3_ Heterostructure

**Figure 10:**
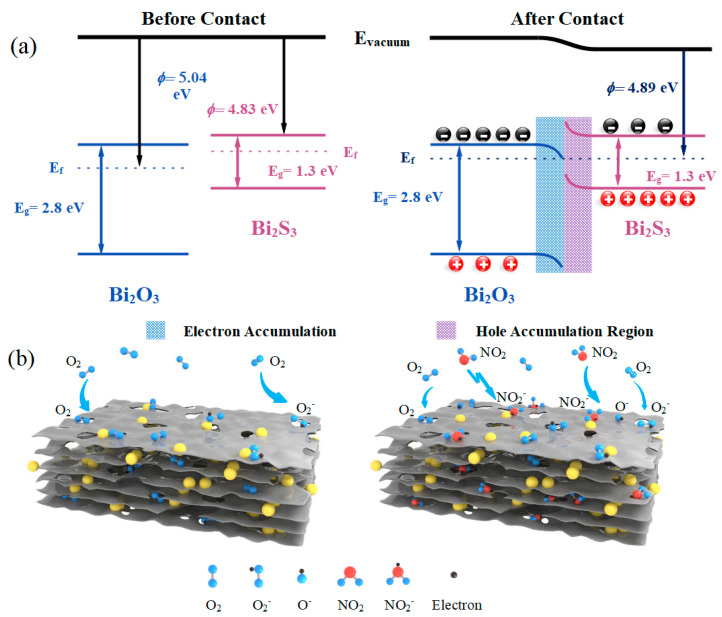
Schematic diagrams of the sensitization mechanisms: (**a**) bandgap structure of Bi_2_S_3_, Bi_2_O_3_, and Bi_2_S_3_/Bi_2_O_3_ hybrid materials and (**b**) the related gas-sensitive procedure.

The enhanced gas-sensitive characteristics of the Bi_2_S_3_/Bi_2_O_3_ heterostructure-based sensor to NO_2_ are primarily assigned to the effect of electron sensitization. The contact potential differences (CPDs) of the pristine Bi_2_S_3_, Bi_2_S_3_/Bi_2_O_3_ hybrid materials, and Bi_2_O_3_ were measured using the single-point mode of KPFM. A gold-plated cantilever with a diameter of 2 mm was adopted as the probe tip. The vibration frequency, data acquisition rate, and bias voltage were set at 79 Hz, 13,500 Hz, and ±7 V, respectively. The work function of the tip and the measured CPDs of the materials were utilized to calculate their corresponding work functions, which were found to be 4.83 eV, 4.89 eV, and 5.04 eV, respectively (see Figure S5). Since both Bi_2_S_3_ and Bi_2_O_3_ are N-type semiconductors, an N-N homojunction with the type-II staggered bandgap is formed as shown in Figure 10a [49,59]. The main carrier electrons are transferred from the conduction band of Bi_2_S_3_ with a relatively narrow bandgap to that of Bi_2_O_3_, which is caused by the concentration gradient. Meanwhile, the holes migrate to the valence band of Bi_2_S_3_ in the opposite direction until the Fermi level reaches dynamic equilibrium. The accumulation of electrons and holes on each side of the heterostructure generates the built-in electric field, thus achieving effective separation of the carriers. The increased electron content at the interface of the heterostructure facilitates greater oxygen molecule ($O_{2(ads)}$) adsorption, increasing the content of chemisorbed oxygen ions ($O_{2(ads)}^{-}$) on the surface of the Bi_2_S_3_/Bi_2_O_3_ hybrid materials. The XPS analysis findings support this observation. Subsequently, the introduced NO_2_ molecules directly capture surface electrons of hybrid materials through physisorption [60]. The redox interaction between NO_2_ molecule and $O_{2(ads)}^{-}$ generates ${NO}_{2(ads)}^{-}$ and $O_{\left(ads\right)}^{-}$ ions, which can further trap additional electrons through chemisorption [61,62]. According to Equations (7) and (8), the combination of both sensing processes leads to the narrowing of the conductive channels in the hybrid materials, increases the barrier height, and aggravates the degree of energy band bending. As a result, a broader range of resistance changes is produced, and the sensitivity of the Bi_2_S_3_/Bi_2_O_3_ heterostructure-based sensor is improved. Figure 10b describes the schematic diagram of gas-sensitive procedure, and the equations of related reactions are as follows:

(5) $$O_{2(gas)}\to O_{2(ads)}$$

(6) $$O_{2(ads)}+e^{-}\to O_{2(ads)}^{-}$$

(7) $${NO}_{2(gas)}+e^{-}\to {NO}_{2(ads)}^{-}$$

(8) $${NO}_{2(gas)}+O_{2(ads)}^{-}+{2e}^{-}\to {NO}_{2(ads)}^{-}+{2O}_{\left(ads\right)}^{-}$$

#### 3.3.2. Roles of Morphological Structure

The particular morphological structure also contributes significantly to improving the gas-sensitive properties of the Bi_2_S_3_/Bi_2_O_3_ heterostructure-based sensor. In the SEM image (see Figure 4b), the surface of the Bi_2_S_3_/Bi_2_O_3_ hybrid materials exhibits the unique mesoporous structure, which could be attributed to two aspects. Since the radius of the O atom is smaller than that of the S atom, the primitive cell volume of Bi_2_O_3_ (~81.07 Å^3^) formed after thermal oxidation is only about 16.05% of that of Bi_2_S_3_ (~505.06 Å^3^), resulting in the lattice distortion and the local collapse of hybrid materials [63,64]. On the other hand, the Bi_2_O_3_ nanoparticles were found to be rich in oxygen vacancies, and the proportion of oxygen vacancies was estimated to be approximately 25.27% by EPR and XPS measurements. This is also the decisive factor for the formation of the mesoporous structure. Owing to this morphological characteristic, the specific surface area of the Bi_2_S_3_/Bi_2_O_3_ hybrid materials increased by approximately 8.1-fold. Moreover, the mesoporous structure facilitates the thorough permeation of O_2_ and NO_2_ molecules, while the oxygen vacancies supply more active sites for gas molecule attachment. Compared to the pristine Bi_2_S_3_, the content of $O_{2(ads)}^{-}$ on the surface of hybrid materials was increased by about 3.05-fold. Thus, the effectiveness of the Bi_2_S_3_/Bi_2_O_3_ heterostructure-based sensor in detecting low-concentration NO_2_ was significantly improved.

#### 3.3.3. DFT Theoretical Calculation

**Figure 11:**
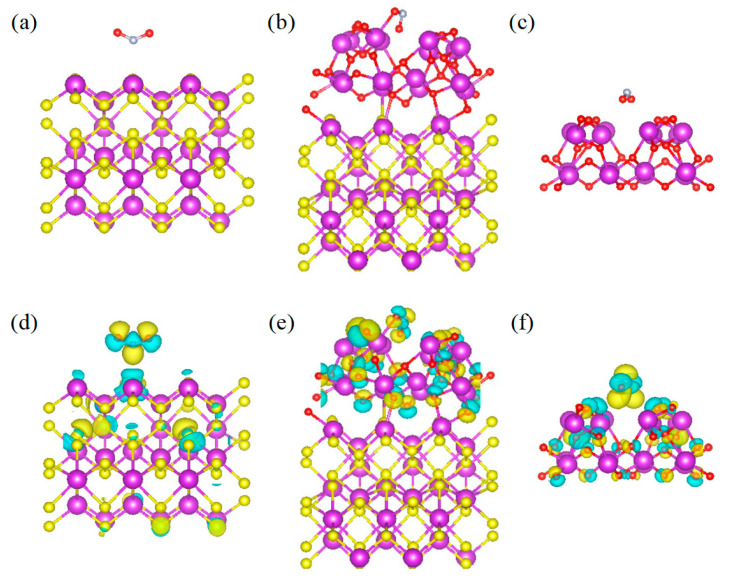
Adsorption structures of NO_2_ adsorbed on (**a**) Bi_2_S_3_, (**b**) Bi_2_S_3_/Bi_2_O_3_ heterostructure, and (**c**) Bi_2_O_3_, and the charge density difference in (**d**) Bi_2_S_3_, (**e**) Bi_2_S_3_/Bi_2_O_3_ heterostructure, and (**f**) Bi_2_O_3_. (the yellow region represents electron accumulation, while the cyan region stands for the electron depletion).

To deeply reveal the sensitization mechanisms, the gas-sensitive processes and electron transfer characteristics of pristine Bi_2_S_3_, Bi_2_S_3_/Bi_2_O_3_ hybrid materials, and Bi_2_O_3_ in interaction with NO_2_ molecules were investigated utilizing DFT calculations. The adsorption sites of NO_2_ molecules on various structures were explored, and the optimized structures for NO_2_ adsorption are shown in Figure 11a–c. In Figure 11b, it could be observed that a new Bi-O bond with the length of 2.41 Å was formed between the NO_2_ molecule and the Bi_2_S_3_/Bi_2_O_3_ heterostructure, which constitutes a stable adsorption structure. The corresponding binding energies of the adsorption sites are shown in Table S2. The adsorption energy was calculated to be approximately −1.26 eV according to Equation (3) mentioned above, indicating that the Bi_2_S_3_/Bi_2_O_3_ heterostructure has strong adsorption capacity for NO_2_. In contrast, the interaction of NO_2_ molecules with the surfaces of Bi_2_S_3_ and Bi_2_O_3_ is primarily governed by relatively weak van der Waals forces, exhibiting typical characteristics of physisorption. The corresponding adsorption energies are approximately −0.21 eV and −0.39 eV, while the distances between NO_2_ molecules and the adsorption sites are measured to be 2.97 Å and 2.81 Å, respectively. Moreover, the electron transfer properties were analyzed, and the related charge density differences are shown in Figure 11d–f. The yellow electron cloud represented electron aggregation, and the cyan area reflected electron depletion. The Bader charge calculations confirm that the electron transfer amounts in the adsorption structures of Bi_2_S_3_, Bi_2_S_3_/Bi_2_O_3_ heterostructure, and Bi_2_O_3_ are −0.17 e, −0.67 e, and −0.08 e, respectively. Evidently, NO_2_ molecules capture numerous electrons from the heterostructure, resulting in the greatest change in resistance. This illustrates that the Bi_2_S_3_/Bi_2_O_3_ heterostructure-based sensor possesses the optimal sensitivity to NO_2_, as demonstrated by the experimental results. The adsorption energies, electron transfer amounts, and the distances between NO_2_ molecules and the adsorption sites are summarized in Table 2.

**Figure 12:**
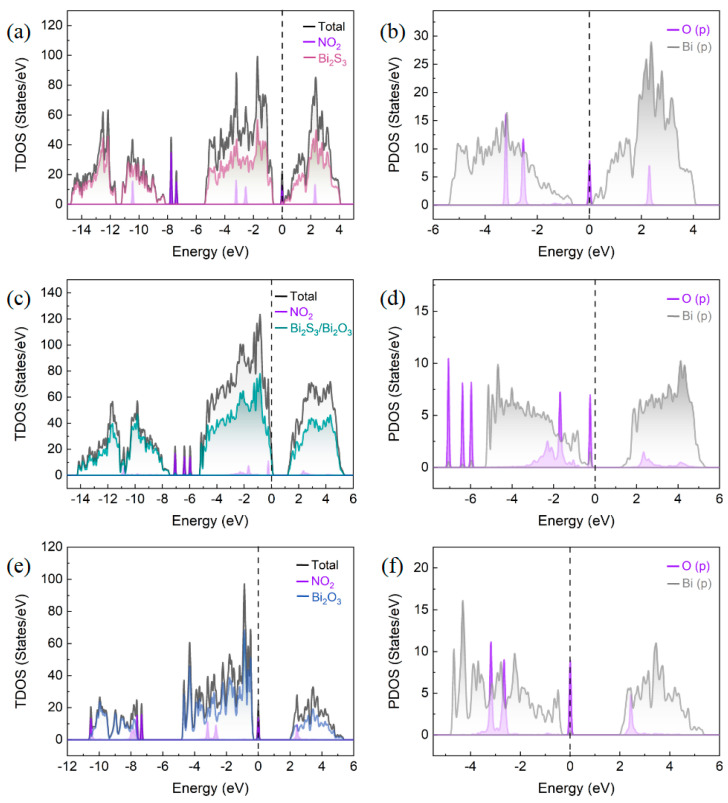
The TDOS and PDOS of NO_2_ adsorbed on (**a**,**b**) Bi_2_S_3_, (**c**,**d**) Bi_2_S_3_/Bi_2_O_3_ heterostructure, and (**e**,**f**) Bi_2_O_3_.

Figure 12 displays the DOS for the adsorption structures of Bi_2_S_3_, the Bi_2_S_3_/Bi_2_O_3_ heterostructure, and Bi_2_O_3_ in the presence of NO_2_ molecules. It can be found that all the structures retain the semiconductor properties with bandgaps after the adsorption of NO_2_ molecules. The total density of states (TDOS) of the three different structures reflects that a new characteristic peak occurs around the Fermi level (0 eV) after NO_2_ molecule adsorption. In particular, compared to the adsorption structures of Bi_2_S_3_ and Bi_2_O_3_, the TDOS of the Bi_2_S_3_/Bi_2_O_3_ heterostructure exhibits the most pronounced peak intensity in the range of −0.1 to −0.4 eV (the position of the NO_2_ characteristic peak) as depicted in Figure 12c, suggesting that the number of electron orbits in this region is the largest. It implies that the heterostructure could provide more electrons to participate in the interaction with NO_2_ molecules. Meanwhile, the comparison of the projected density of states (PDOS) describes that the overlapping areas of the p orbits of O atoms in NO_2_ and the p orbits of Bi atoms on the Bi_2_S_3_/Bi_2_O_3_ heterostructure surface are enlarged in both the conduction band (from 1.9 eV to 5.0 eV) and the valence band (from −0.7 eV to −3.7 eV) as shown in Figure 12d, indicating a strong orbital hybridization effect. The enhanced electron delocalization due to orbital convergence redistributes electrons on the Bi_2_S_3_/Bi_2_O_3_ heterostructure surface. As a conclusion, the NO_2_ molecules could capture more electrons from the hybrid materials, thus boosting the gas-sensitive properties of the Bi_2_S_3_/Bi_2_O_3_ heterostructure-based sensor.

## 4. Conclusions

The Bi_2_S_3_/Bi_2_O_3_ hybrid materials with mesoporous structure were prepared utilizing a facile in situ elevated temperature thermal oxidation method. The influence of various oxidation temperatures (300~500 °C) and oxidation times (0~2 h) on the gas sensitivity of the as-fabricated sensor was explored, and the formation mechanism of the mesoporous structure was analyzed in detail. The Bi_2_S_3_/Bi_2_O_3_ heterostructure-based sensor synthesized at 400 °C for 1 h exhibits a response of around 7.85 to 8 ppm NO_2_ at RT, reflecting a 3.5-fold boost compared to the pristine Bi_2_S_3_-based sensor. The response and recovery times are 71 s and 238 s, and the detection limit for low-concentration NO_2_ can reach 0.1 ppm. The sensor was also proven to possess exceptional signal repeatability, gas selectivity, and long-term stability. The response deviation of the sensor remained below 6.6% after 11 weeks of measurement. The significant improvement of the Bi_2_S_3_/Bi_2_O_3_ heterostructure-based sensor can be assigned to electronic sensitization and distinct morphological structure. Furthermore, DFT was employed to calculate the adsorption energies, electron transfer amounts, and DOS for various NO_2_ adsorption structures. It reveals that the orbital hybridization between O atoms in NO_2_ molecules and Bi atoms on the heterostructure surface promotes the electron delocalization, resulting in the redistribution of electrons. Thus, NO_2_ molecules can capture more electrons. This work demonstrates from both theoretical and experimental perspectives that the Bi_2_S_3_/Bi_2_O_3_ hybrid materials with the mesoporous structure have excellent NO_2_ detectability and could serve as a potential material for high-performance and low-energy-consumption gas sensors.

## Figures and Tables

**Table 1:** The comparison of NO_2_ sensing performance of the prepared Bi_2_S_3_/Bi_2_O_3_ heterostructure-based sensor with the previously reported Bi_2_S_3_-based sensors at RT.

Materials	Gas	Temp. (°C)	Conc. (ppm)	$\mathrm{Responses}\;(R_{gas}/R_{air)}$	T_res_/T_res_ (s)	LOD (ppm)	Refs.
Bi_2_S_3_	NO_2_	RT	100	1.39	14/257	1	[21]
Bi_2_S_3_	NO_2_	RT	10	12.2	39/696	2	[25]
Au/Bi_2_S_3_	NO_2_	RT	5	5.6	21/371	0.25	[57]
CuS/Bi_2_S_3_	NO_2_	RT	10	3.4	18/338	0.5	[29]
Bi_2_S_3_/Bi_2_O_3_	NO_2_	RT	10	1.28	26.6/69.9	0.1	[58]
Bi_2_S_3_/Bi_2_O_3_	NO_2_	RT	8	7.85	71/238	0.1	this work

T_res_/T_rec_ = response/recovery time; LOD= limit of detection; Temp. = temperature; Conc. = concentration.

**Table 2:** The adsorption energies ($E_{ads}$), electron transfer amounts (∆Q), and the distances (d) between NO_2_ and corresponding adsorption sites.

Configurations	$E_{ads}$ (eV)	∆Q (e)	d (Å)
Bi_2_S_3_/Bi_2_O_3_	−1.26	−0.67	2.41
Bi_2_S_3_	−0.21	−0.17	2.97
Bi_2_O_3_	−0.39	−0.08	2.81

## Data Availability

The data presented in this study are available on request from the corresponding author.

## Supplementary Materials

The following supporting information can be downloaded at: https://www.mdpi.com/article/10.3390/s25123612/s1; Figure S1: XPS full survey spectrums of the pristine Bi_2_S_3_, Bi_2_S_3_/Bi_2_O_3_ hybrid materials and Bi_2_O_3_; Figure S2: I–V polarization curves of the pristine Bi_2_S_3_, Bi_2_S_3_/Bi_2_O_3_ heterostructure, and Bi_2_O_3_-based sensors at RT; Figure S3: The response curves of the Bi_2_S_3_/Bi_2_O_3_ heterostructure-based sensor under various humidities to 8 ppm NO_2_; Figure S4: The resistance curves of the Bi_2_S_3_/Bi_2_O_3_ heterostructure-based sensor under various humidities to 8 ppm NO_2_; Figure S5: The work functions of the (a) Bi_2_S_3_, (b) Bi_2_S_3_/Bi_2_O_3_ hybrid materials and (c) Bi_2_O_3_; Table S1: The relative contents of the O_C_ (chemisorbed oxygen), O_L_ (lattice oxygen), and O_V_ (oxygen vacancy) in the synthesized materials; Table S2: The binding energies ($E_{b}$) of NO_2_ molecule at various adsorption sites on the surface of Bi_2_S_3_/Bi_2_O_3_ heterostructure.

## References

[1] Zhao X., Xu Z., Zhang Z., Liu J., Yan X., Zhu Y., Attfield J.P., Yang M. (2025). Titanium Nitride Sensor for Selective NO_2_ Detection. Nat. Commun..

[2] Zhang Y., Jiang Y., Yuan Z., Liu B., Zhao Q., Huang Q., Li Z., Zeng W., Duan Z., Tai H. (2023). Synergistic Effect of Electron Scattering and Space Charge Transfer Enabled Unprecedented Room Temperature NO_2_ Sensing Response of SnO_2_. Small.

[3] Chen Y., Li Z., Tang T., Cheng Y., Cheng L., Wang X., Haidry A.A., Jannat A., Ou J.Z. (2024). Room-Temperature Optoelectronic NO_2_ Sensing Using Two-Dimensional Gallium Oxyselenides. ACS Appl. Nano Mater..

[4] Sun S., Li X., Sun Y., Wang N., Huang B., Li X. (2025). Fabrication of TeNT/TeO_2_ Heterojunction Based Sensor for Ultrasensitive Detection of NO_2_. J. Hazard. Mater..

[5] Wang B., Gao X., He J., Xiao Y., Liu Y., Jia X., Zhang K., Wang C., Sun P., Liu F. (2024). Room-Temperature Ppb-Level NO_2_ Sensor Based on Three-Dimensional Mo_2_CT_x_ Nano-Crumpled Spheres. Sens. Actuators B Chem..

[6] Du H., Zhang Z., Jiang X., Wang J., Yi W., Li X., Chu J. (2023). Enhancement of NO_2_ Gas Sensing Properties of Polypyrrole by Polarization Doping with DBS: Experimental and DFT Studies. ACS Appl. Mater. Interfaces.

[7] Hu Y., Li T., Zhang J., Guo J., Wang W., Zhang D. (2022). High-Sensitive NO_2_ Sensor Based on p-NiCo_2_O_4_/n-WO_3_ Heterojunctions. Sens. Actuators B Chem..

[8] Ding Y., Guo X., Kuang D., Hu X., Zhou Y., He Y., Zang Z. (2021). Hollow Cu_2_O Nanospheres Loaded with MoS_2_/Reduced Graphene Oxide Nanosheets for Ppb-Level NO_2_ Detection at Room Temperature. J. Hazard. Mater..

[9] Zhang W., Wang W., Ge Y., Sun L., Zhou C., Sun Y., Hu J. (2024). SnS_2_/Mo_4/3_B_2_ MBene Microcomposites for Highly Sensitive NO_2_ Sensor at Room Temperature. Sens. Actuators B Chem..

[10] Li Q., Zeng W., Li Y. (2022). Metal Oxide Gas Sensors for Detecting NO_2_ in Industrial Exhaust Gas: Recent Developments. Sens. Actuators B Chem..

[11] Li Z., Liu X., Zhou M., Zhang S., Cao S., Lei G., Lou C., Zhang J. (2021). Plasma-Induced Oxygen Vacancies Enabled Ultrathin ZnO Films for Highly Sensitive Detection of Triethylamine. J. Hazard. Mater..

[12] Lim K., Jo Y.M., Yoon J.W., Kim J.S., Lee D.J., Moon Y.K., Yoon J.W., Kim J.H., Choi H.J., Lee J.H. (2021). A Transparent Nanopatterned Chemiresistor: Visible-Light Plasmonic Sensor for Trace-Level NO_2_ Detection at Room Temperature. Small.

[13] Majhi S.M., Mirzaei A., Kim H.W., Kim S.S., Kim T.W. (2021). Recent Advances in Energy-Saving Chemiresistive Gas Sensors: A Review. Nano Energy.

[14] Goswami P., Gupta G. (2022). Recent Progress of Flexible NO_2_ and NH_3_ Gas Sensors Based on Transition Metal Dichalcogenides for Room Temperature Sensing. Mater. Today Chem..

[15] Tyagi D., Wang H., Huang W., Hu L., Tang Y., Guo Z., Ouyang Z., Zhang H. (2020). Recent Advances in Two-Dimensional-Material-Based Sensing Technology toward Health and Environmental Monitoring Applications. Nanoscale.

[16] Galstyan V., Moumen A., Kumarage G.W.C., Comini E. (2022). Progress towards Chemical Gas Sensors: Nanowires and 2D Semiconductors. Sens. Actuators B Chem..

[17] Rohaizad N., Mayorga-Martinez C.C., Fojtů M., Latiff N.M., Pumera M. (2021). Two-Dimensional Materials in Biomedical, Biosensing and Sensing Applications. Chem. Soc. Rev..

[18] Sysoev V.V., Lashkov A.V., Lipatov A., Plugin I.A., Bruns M., Fuchs D., Varezhnikov A.S., Adib M., Sommer M., Sinitskii A. (2022). UV-Light-Tunable p-/n-Type Chemiresistive Gas Sensors Based on Quasi-1D TiS_3_ Nanoribbons: Detection of Isopropanol at Ppm Concentrations. Sensors.

[19] Zhu L., Zhang J., Wang J., Liu J., Yan W. (2024). Hierarchical Heterojunctions of Metal Sulfide WS_2_ Nanosheets/Metal Oxide In_2_O_3_ Nanofibers for an Efficient Detection of Formaldehyde. Nanomaterials.

[20] Meng T.Y., Hu K.S., Hsueh T.J. (2025). A 2D Tin Disulfide Nanosheet Gas Sensor for Hydrogen Sulfide. IEEE Sens. J..

[21] Harke S.S., Jadhav Y., Patil V.B., Gurnani C. (2025). Facile Solution-Processed Deposition of Bi_2_S_3_ Nanostructures for a Highly Sensitive and Selective Room-Temperature NO_2_ Sensor. ACS Appl. Electron. Mater..

[22] Russ T., Hu Z., Li L., Zhou L., Liu H., Weimar U., Barsan N. (2022). In Operando Investigation of the Concentration Dependent NO_2_ Sensing Mechanism of Bi_2_S_3_ Nanorods at Low Temperatures and the Interference of O_3_. ACS Sens..

[23] Yang Y., Liu C., Wang Y., Hao J. (2024). Nanorods Assembled Hierarchical Bi_2_S_3_ for Highly Sensitive Detection of Trace NO_2_ at Room Temperature. Chemosensors.

[24] Yang Y., Xin T., Liu C., Zhang T., Hao W., Wang Y., Hao J. (2023). Urchin-like Bi_2_S_3_ Nanostructures with Rich Sulfur Vacancies for Ppb-Level NO_2_ Sensing. J. Alloys Compd..

[25] Kan H., Yang W., Guo Z., Li M. (2024). Highly Sensitive Room-Temperture NO_2_ Gas Sensor Based on Bi_2_S_3_ Nanorods. J. Mater. Sci. Mater. Electron..

[26] Li H., Huang H., Huang W., Zhang X., Hai G., Lai F., Zhu T., Bai S., Zhang N., Liu T. (2024). Interfacial Accumulation and Stability Enhancement Effects Triggered by Built-in Electric Field of SnO_2_/LaOCl Nanofibers Boost Carbon Dioxide Electroreduction. Small.

[27] Kim Y., Lee S., Song J.G., Ko K.Y., Woo W.J., Lee S.W., Park M., Lee H., Lee Z., Choi H. (2020). 2D Transition Metal Dichalcogenide Heterostructures for P- and n-Type Photovoltaic Self-Powered Gas Sensor. Adv. Funct. Mater..

[28] Yu M., Li J., Yin D., Zhou Z., Wei C., Wang Y., Hao J. (2024). Enhanced Oxygen Anions Generation on Bi_2_S_3_/Sb_2_S_3_ Heterostructure by Visible Light for Trace H_2_S Detection at Room Temperature. J. Hazard. Mater..

[29] Chen X., Wang T., Shi J., Lv W., Han Y., Zeng M., Yang J., Hu N., Su Y., Wei H. (2022). A Novel Artificial Neuron-Like Gas Sensor Constructed from CuS Quantum Dots/Bi_2_S_3_ Nanosheets. Nano-Micro Lett..

[30] Liu L., Gao Y., Hu Q. (2023). Controllable Epitaxial-like Growth of Bi_2_S_3_/MoS_2_ Hybrid Aerogel Nanostructures for Sensitive NO_2_ Detection at Room Temperature. ACS Appl. Nano Mater..

[31] Yang Y., Zhu M., Zhang H., Wang B., Chen C., Li J., Wang Y., Hao J. (2024). Room Temperature Gas Sensor Based on rGO/Bi_2_S_3_ Heterostructures for Ultrasensitive and Rapid NO_2_ Detection. Chem. Eng. J..

[32] Zhao X., Zhi M., Hang D., Ren Q., Zhang P., Chen C., Chen Q., Li Q., Zhang Z., Yan J. (2023). Ultrasensitive NO_2_ Gas Sensor Based on MoS_2_ Modified Urchin-like Bi_2_S_3_ Heterojunction. Phys. E Low-Dimens. Syst. Nanostructures..

[33] Kresse G., Furthmüller J. (1996). Efficiency of Ab-Initio Total Energy Calculations for Metals and Semiconductors Using a Plane-Wave Basis Set. Comput. Mater. Sci..

[34] Kresse G., Furthmüller J. (1996). Efficient Iterative Schemes for Ab Initio Total-Energy Calculations Using a Plane-Wave Basis Set. Phys. Rev. B Condens. Matter Mater. Phys..

[35] Perdew J.P., Burke K., Ernzerhof M. (1996). Generalized Gradient Approximation Made Simple. Phys. Rev. Lett..

[36] Li X., Sun S., Wang N., Huang B., Li X. (2024). SnTe/SnSe Heterojunction Based Ammonia Sensors with Excellent Withstand to Ambient Humidities. Small.

[37] Xing H., Li X., Sun S., Huang B., Li X. (2024). Low-Temperature NO_2_ Sensor Based on γ-In_2_Se_3_/In_2_O_3_ Nanoflower Heterojunction. Sens. Actuators B Chem..

[38] Lan M., Dong X., Zheng N., Zhang X., Wang Y., Zhang X. (2023). In-Situ Construction of Novel Sulfur-Vacancy-Rich Bi/Bi_2_S_3_/SnS_2_ Z-Scheme Heterostructure Photocatalysts for Efficient Cr(VI) Reduction and Nitrogen Fixation. J. Mater. Sci. Technol..

[39] Chen D.N., Wang G.Q., Mei L.P., Feng J.J., Wang A.J. (2023). Dual II-Scheme Nanosheet-like Bi_2_S_3_/Bi_2_O_3_/Ag_2_S Heterostructures for Ultrasensitive PEC Aptasensing of Aflatoxin B1 Coupled with Catalytic Signal Amplification by Dendritic Nanorod-like Au@Pd@Pt Nanozyme. Biosens. Bioelectron..

[40] Wang X., Du C., Yu Y., Li W., Li T., Wang S., Mao S., Wang Y., Zhao J., Xiong C. (2024). Efficient Photocatalytic Degradation with a Lattice-Matched α-Bi_2_O_3_/Co_3_O_4_ Z-Scheme Heterojunction: An Integrated Experimental and DFT Study. J. Water Process Eng..

[41] Cheng X., Xiao X., Wang F., Lu T., Zhang Y. (2024). Heterojunctions Based on BiOBr Nanosheets Decorated on α-Bi_2_O_3_ for Photodegradation of Rhodamine B. ACS Appl. Nano Mater..

[42] Tang Y., Gan S., Zhong L., Sun Z., Xu L., Liao C., Lin K., Cui X., He D., Ma Y. (2022). Lattice Proton Intercalation to Regulate WO_3_-Based Solid-Contact Wearable PH Sensor for Sweat Analysis. Adv. Funct. Mater..

[43] Bai Y., Fu H., Yang X., Xiong S., Li S., An X. (2023). Conductometric Isopropanol Gas Sensor: Ce-Doped In_2_O_3_ Nanosheet-Assembled Hierarchical Microstructure. Sens. Actuators B Chem..

[44] Zhang Q., Xie G., Duan M., Liu Y., Cai Y., Xu M., Zhao K., Tai H., Jiang Y., Su Y. (2023). Zinc Oxide Nanorods for Light-Activated Gas Sensing and Photocatalytic Applications. ACS Appl. Nano Mater..

[45] Cheng L., Li Y., Sun G., Cao J., Wang Y. (2023). Modification of Bi_2_O_3_ on ZnO Porous Nanosheets-Assembled Architecture for Ultrafast Detection of TEA with High Sensitivity. Sens. Actuators B Chem..

[46] Kashmery H.A., El-Hout S.I. (2023). Bi_2_S_3_/Bi_2_O_3_ Nanocomposites as Effective Photocatalysts for Photocatalytic Degradation of Tetracycline under Visible-Light Exposure. Opt. Mater..

[47] Wang J., Chen J., Liu F., Jia M., Zhang Z., Liu M., Jiang L. (2022). Renewable WO_3_/Bi_2_O_3_ Heterojunction for Photoelectrochemical and Visual Dual-Mode Detection of Hydrogen Sulfide. Sens. Actuators B Chem..

[48] Guang Q., Huang B., Yu J., Bonyani M., Moaddeli M., Kanani M., Mirzaei A., Kim H.W., Kim S.S., Li X. (2023). PtS-Decorated WS_2_ Microflakes Based Sensors for Selective Ammonia Detection at Room Temperature. Sens. Actuators B Chem..

[49] Ma Y., Jiang X., Sun R., Yang J., Jiang X., Liu Z., Xie M., Xie E., Han W. (2020). Z-Scheme Bi_2_O_2.33_/Bi_2_S_3_ Heterojunction Nanostructures for Photocatalytic Overall Water Splitting. Chem. Eng. J..

[50] Cheng D., Wu H., Feng C., Ding Y., Mei H. (2022). Bifunctional Photoelectrochemical Sensor Based on Bi/Bi_2_S_3_/BiVO_4_ for Detecting Hexavalent Chromium and Hydrogen Peroxide. Sens. Actuators B Chem..

[51] Ou L.X., Liu M.Y., Zhu L.Y., Zhang D.W., Lu H.L. (2022). Recent Progress on Flexible Room-Temperature Gas Sensors Based on Metal Oxide Semiconductor. Nano-Micro Lett..

[52] Zheng Y., Cui X., Zhou Y., Zhang H., Cao L., Gao L., Yin H., Ai S. (2022). MXene Enhanced Photoactivity of Bi_2_O_3_/Bi_2_S_3_ Heterojunction with G-Wire Superstructure for Photoelectrochemical Detection of TET1 Protein. ACS Sens..

[53] Ikram M., Liu L., Lv H., Liu Y., Ur Rehman A., Kan K., Zhang W.J., He L., Wang Y., Wang R. (2019). Intercalation of Bi_2_O_3_/Bi_2_S_3_ Nanoparticles into Highly Expanded MoS_2_ Nanosheets for Greatly Enhanced Gas Sensing Performance at Room Temperature. J. Hazard. Mater..

[54] Li H., Jing P., He C., Pan Z., Liu J., Cui Y., Wang J. (2023). Interfacial Configuration and Mechanism Insights of an All-Solid-State Z-Scheme BaTiO_3_/Bi/Bi_2_O_3_ Heterojunctions for Rapid Removal of Tetracycline Antibiotics. Appl. Surf. Sci..

[55] Chang J., Liang W., Wang W., Wu D., Jiang K., Wang G., Xu F., Gao Z. (2022). Oxygen Vacancies Enriched Bi_2_O_3_ as High Capacity and High Rate Negative Material for Aqueous Alkali Battery. Appl. Surf. Sci..

[56] Li X., Zhang Z., Sun S., Wang N., Huang B., Li X. (2024). Hierarchically In_2_S_3_@In_2_O_3_ Nanorods Heterojunctions for Enhanced NO_2_ Sensing at Lower Operating Temperature. Sens. Actuators B Chem..

[57] Chen X., Shi J., Wang T., Zheng S., Lv W., Chen X., Yang J., Zeng M., Hu N., Su Y. (2022). High-Performance Wearable Sensor Inspired by the Neuron Conduction Mechanism through Gold-Induced Sulfur Vacancies. ACS Sens..

[58] Wang X., Chen Y., Zeng Z., Yan M., Jia X., Hu P., Xu J., Xue Z., Xu J. (2024). Tailoring the Injection Action of Oxygen over Top-Surface of Bismuth Sulfide to Change Reactive Electron Transfer Path for Flexible NO_2_ Sensors. Mater. Sci. Eng. R. Rep..

[59] Chang F., Peng S., Yan W., Yang C., Li S., Liu X. (2021). A Novel and Facile Procedure to Decorate Bi_2_O_3_ with Bi_2_S_3_ Nanocrystals: Composites Synthesis, Analyses, and Photocatalytic Performance Assessment. Colloids Surf. A Physicochem. Eng. Asp..

[60] Gasso S., Sohal M.K., Mahajan A. (2022). MXene Modulated SnO_2_ Gas Sensor for Ultra-Responsive Room-Temperature Detection of NO_2_. Sens. Actuators B Chem..

[61] Bai H., Guo H., Wang J., Dong Y., Liu B., Xie Z., Guo F., Chen D., Zhang R., Zheng Y. (2021). A Room-Temperature NO_2_ Gas Sensor Based on CuO Nanoflakes Modified with rGO Nanosheets. Sens. Actuators B Chem..

[62] Hau H.H., Duong T.T.H., Man N.K., Thi Viet Nga T., Thi Xuan C., Thi Thanh Le D., Van Toan N., Hung C.M., Van Duy N., Van Hieu N. (2021). Enhanced NO_2_ Gas-Sensing Performance at Room Temperature Using Exfoliated MoS_2_ Nanosheets. Sens. Actuators A Phys..

[63] Yakout S.M. (2020). New Efficient Sunlight Photocatalysts Based on Gd, Nb, V and Mn Doped Alpha-Bi_2_O_3_ Phase. J. Environ. Chem. Eng..

[64] Motaung M.P., Onwudiwe D.C., Lei W. (2021). Microwave-Assisted Synthesis of Bi_2_S_3_ and Sb_2_S_3_ Nanoparticles and Their Photoelectrochemical Properties. ACS Omega.

