# Supplementary material for: Mesoporous Bi2S3/Bi2O3 Heterostructure-Based Sensors for Sub-ppm NO2 Detection at Room Temperature
Source: Sensors (Basel). 2025 Jun 9;25(12):3612. doi: 10.3390/s25123612 (PMC12196864; doi:10.3390/s25123612)
Supplement: Supplementary file 1 [file sensors-25-03612-s001.zip › sensors-3650536-supplementary.pdf]

Supporting Information

Mesoporous Bi_2S_3/Bi_2O_3 Heterostructure-based Sensors for Sub-ppm NO_2 Detection at Room Temperature

Wei Liu[1], *Jiashuo Chen*[1], *Ding Gu*[2]*, *Shupeng Sun*[3], *Xinlei Li*[3], *Xiaogan Li*[3]

[1]School of Electronics and Information Engineering, Nanjing University of Information Science and Technology, Nanjing 210044, China

[2]School of Microelectronics and Control Engineering, Changzhou University, Changzhou 213164, China

[3]School of Integrated Circuits, Dalian University of Technology, Dalian 116024, China

*The authors all correspondence should be addresses to: E-mail: guding0815@cczu.edu.cn

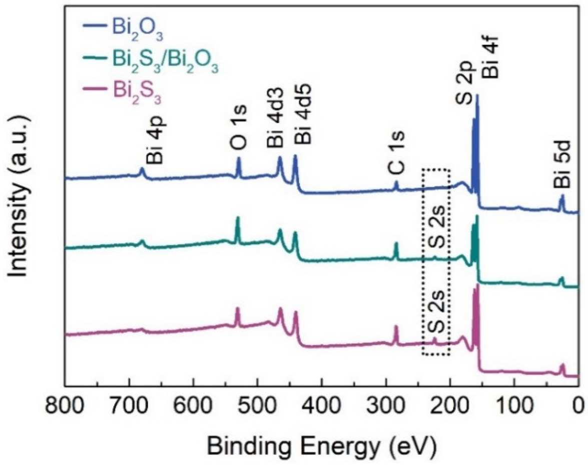

Figure S1. XPS full survey spectrums of the pristine Bi_2S_3, Bi_2S_3/Bi_2O_3 hybrid materials and Bi_2O_3.

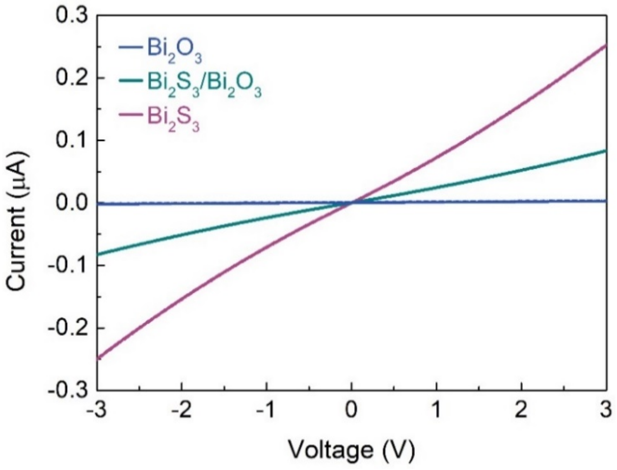

Figure S2. I-V polarization curves of the pristine Bi_2S_3, Bi_2S_3/Bi_2O_3 heterostructure, and Bi_2O_3-based sensors at RT.

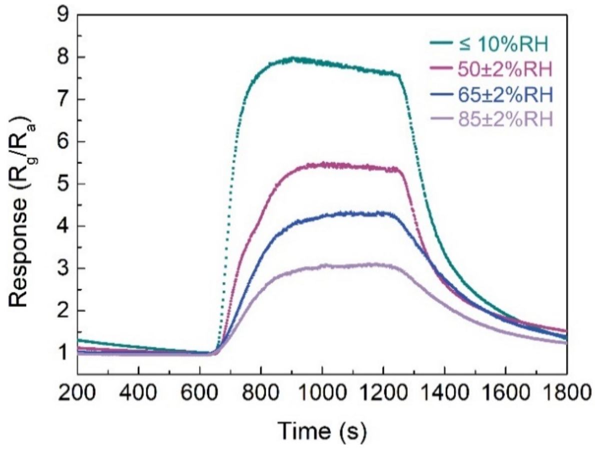

Figure S3. The response curves of the Bi_2S_3/Bi_2O_3 heterostructure-based sensor under various humidities to 8 ppm NO_2.

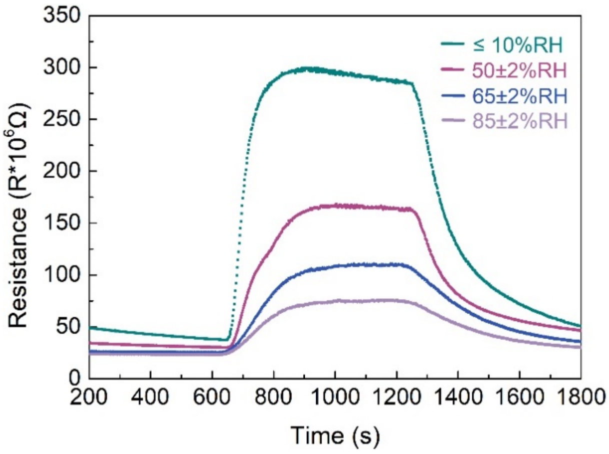

Figure S4. The resistance curves of the Bi_2S_3/Bi_2O_3 heterostructure-based sensor under various humidities to 8 ppm NO_2.

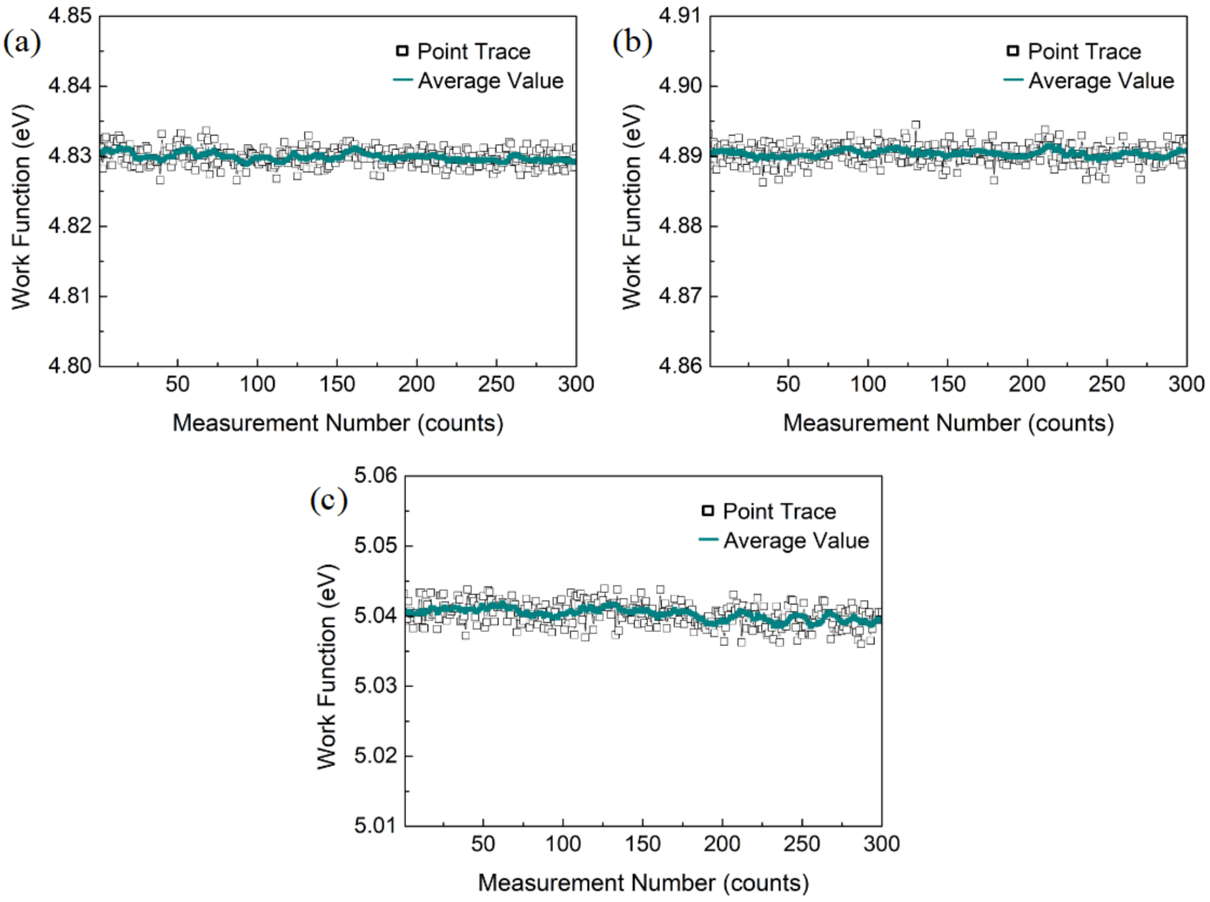

Figure S5. The work functions of the (a) Bi_2S_3, (b) Bi_2S_3/Bi_2O_3 hybrid materials and (c) Bi_2O_3.

Table S1. The relative contents of the O_C (chemisorbed oxygen), O_L (lattice oxygen), and O_V (oxygen vacancy) in the synthesized materials.

Materials	Integral areas of O 1s	Integral areas of O_C	Integral areas of O_L	Integral areas of O_V	Relative content of O_C (compared to Bi_2S_3)	Relative content of O_V (compared to O 1s)
Bi_2S_3	21093.12	21093.12	/	/	1	/
Bi_2S_3/Bi_2O_3	158934.91	64389.88	82536.42	40164.39	3.05	25.27%
Bi_2O_3	168235.57	23936.02	132472.20	18994.32	1.13	11.29%

Table S2. The binding energies (E_b) of NO_2 molecule at various adsorption sites on the surface of Bi_2S_3/Bi_2O_3 heterostructure.

Adsorption Sites	E_b (eV)	E_{ads} (eV)
1	-2.98	-1.26
2	-2.62	-0.37
3	-2.95	-0.08
4	-2.65	-1.26
5	-2.51	-1.16
6	-2.68	-1.15

Adsorption Sites:

1. NO_2 was placed horizontally with the N atom facing upwards, and located in the center of the surface.

2. NO_2 was placed horizontally with the N atom facing down, and located in the center of the surface.

3. NO_2 was placed vertically in the center of the surface.

4. NO_2 was placed horizontally with the N atom facing upwards, and located above the O atom of Bi_2O_3.

5. NO_2 was placed horizontally with the N atom facing down, and located above the O atom of Bi_2O_3.

6. NO_2 was placed vertically above the O atom of Bi_2O_3.
